# Mechanical Ventilation Weaning in Inclusion Body Myositis: Feasibility of Isokinetic Inspiratory Muscle Training as an Adjunct Therapy

**DOI:** 10.1155/2014/902541

**Published:** 2014-07-24

**Authors:** Leonardo Cordeiro de Souza, Josué Felipe Campos, Leandro Possidente Daher, Priscila Furtado da Silva, Alex Ventura, Pollyana Zamborlini do Prado, Daniele Brasil, Debora Mendonça, Jocemir Ronaldo Lugon

**Affiliations:** ^1^Medical Science Post-Graduation Program, Universidade Federal Fluminense, Niterói, RJ, Brazil; ^2^Intensive Care Division, Hospital e Clínica São Gonçalo, São Gonçalo, RJ, Brazil; ^3^Nephrology Division, Department of Medicine, Medical School, Universidade Federal Fluminense, Niterói, RJ, Brazil

## Abstract

Inclusion body myositis is a rare myopathy associated with a high rate of respiratory complications. This condition usually requires prolonged mechanical ventilation and prolonged intensive care stay. The unsuccessful weaning is mainly related to respiratory muscle weakness that does not promptly respond to immunosuppressive therapy. We are reporting a case of a patient in whom the use of an inspiratory muscle-training program which started after a two-week period of mechanical ventilation was associated with a successful weaning in one week and hospital discharge after 2 subsequent weeks.

## 1. Introduction 

In 1971, the term “inclusion body myositis” (IBM) was coined to describe a subset of patients with chronic myositis, whose muscle biopsy showed besides inflammation, muscle fibres containing vacuoles and abnormal filaments, and characteristic inclusions in cytoplasm and nucleus [1]. The inclusion body myositis is grouped with dermatomyositis and polymyositis as idiopathic inflammatory myopathies [2]. The disease is characterized by a proximal myopathy, which progressively leads to functional disability. It is more common in adults in their fifties [1, 2]. Respiratory complications can reach a prevalence of 45%. Weakness/fatigue of respiratory muscles with interstitial pneumonia and dysphagia are the main causes of death [2–4]. The use of mechanical ventilation is required in most cases, but weaning is hardly achieved [4, 5]. We are reporting a case in whom an inspiratory muscle training program (IMT) using an isokinetic electronic load device which started after a two-week period of mechanical ventilation was associated with a successful weaning in one week.

## 2. Case Report 

A 43-year-old male was admitted to the intensive care of the hospital with symmetrical muscle weakness in the pelvic and scapular girdles. He reported a history of Guillain-Barré syndrome in 2005 without motor sequelae. His initial laboratory exams were haemoglobin of 16.1 g/dL; leukocytes of 13,030/mm^3^ with 3% young forms and 13% of lymphocytes; urea of 23 mg/dL; creatinine of 0.34 mg/dL; Na of 131 mEq/L; K of 5.5 mEq/L; Cl of 97 mEq/L; AST of 51 U/L; ALT of 49 U/L; lactate of 1.1 mEq/L; LDL of 926 U/L; CK of 145 U/L; and CRP of 4.6 mg/dL.

He evolved with acute respiratory failure type II (PaO_2_ < 60 mmHg and PaCO_2_ > 45 mmHg) requiring orotracheal intubation and mechanical ventilation that was set on a pressure controlled mode. His cerebrospinal fluid showed the presence of lymphocytes, monocytes, neutrophils, and macrophages, blood cultures were negative for bacterial or fungal agents, and serological tests for toxoplasmosis, herpes, varicella, and HIV were all negative. A deltoid muscle biopsy showed a mononuclear interstitial inflammatory infiltrate with fibres exhibiting rimmed vacuoles, findings that were thought to be compatible with the diagnosis of inclusion body myositis.

During his stay in the ICU, he received a course of intravenous immunoglobulin (0.4 g/kg per day for 5 days) and oral prednisone (1 mg/kg per day) without improvement [4]. In the same period, he developed nosocomial pneumonia and septic shock and was managed with vasoactive amines and intravenous antibiotic therapy (meropenem combined with polymyxin B).

After three unsuccessful weaning trials, the patient was tracheotomised on the 14th day of intubation and transferred to the prolonged ventilation unit. Following hemodynamic stabilization and infection control, azathioprine 150 mg per day [4] was started in association with a inspiratory muscle training (IMT) program using an isokinetic electronic load device (POWERbreathe K-5, London, UK), 2 daily sessions of 30 respiratory cycles as previously described [9], with a load of 30% of the expected maximal inspiratory pressure as described elsewhere [10–12]. Load and intensity were reassessed weekly using commercial software (BreatheLink, POWERbreathe Co., London, UK). During the period of treatment, the patient comfortably remained under pressure support ventilation (frequency < 30). The IMT program was accompanied by an early mobilization protocol (active member exercising in bed followed by resistance exercising against gravity at the bedside, evolving to standing position and cycle ergometer activity) [13].

At the start of the new therapeutic program, the measured respiratory parameters of the patient were static compliance of 55 mL/cm H_2_O, SpO_2_ of 95% (FIO_2_ 35%), breathing frequency of 21 bpm, tidal volume of 0.390 L, an f/Vt index of 54 bpm/L, P0.1 of 4.66 cm H_2_O, MIP of 20 cm H_2_O (17% of the expected value of 118 cm H_2_O), and timed inspiratory effort (TIE) index of 0.54 cm H_2_O/sec (~50% of the expected value). The muscle function evaluation of the patient at the beginning of the training program showed an Medical Research Council (MRC) score of 22 (see Table 1), while the correspondent values for the degree of independence of the patient as assessed by the motor domain of the Functional Independence Measure (FIM) score was 14; see Table 2.

**Figure 1:**
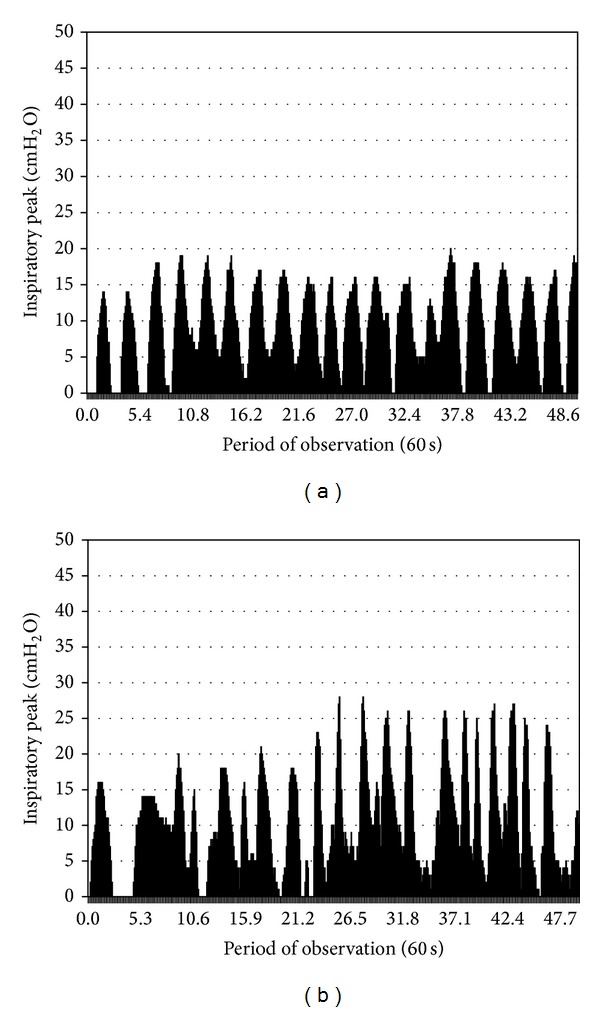
Graphics of inspiratory peaks by TIE index method. (a) At start of program on mechanical ventilation dependence; (b) 14 days after treatment associated with successful weaning.

After two weeks of IMT, the MIP increased to 28 cm H_2_O, the TIE index to 1.09 cm H_2_O/sec, and a partial respiratory independence was achieved (nocturnal pressure support ventilation was provided) [4]. After four weeks, MIP was 50 cm H_2_O; see Figure 1. By that time, the load employed with the power breath device was 15 cm H_2_O. He progressed to decannulation and was maintained on nocturnal noninvasive ventilation to avoid hypoventilation and carbonarcosis. By the time of hospital discharge, he was under IMT twice a day with a load of 17 cm H_2_O and walking 380 m independently, but under supervision, and climbing a flight of stairs. His MRC score was 52 (see Table 1) and the motor FIM score was 81 (see Table 2). After 6 months of hospital discharge, the patient remains on a regular use of the inspiratory load device and continues to improve his muscle strength. He is now gradually coming back to his daily activities.

## 3. Discussion

We are reporting a case of body inclusion myositis in which the definite diagnosis was obtained early in the course of the disease. Following previous recommendations [1, 3–5], therapy was promptly started with intravenous immunoglobulin and steroids; the maintenance drug chosen was azathioprine. The experience with the treatment of this entity has been disappointing [1, 4, 5]. Unsuccessful weaning represents a major obstacle to patient recovery and is associated with a high rate of respiratory infection and a fatal outcome [2–4, 14]. Although effective in the management of the subjacent process that precipitated the disorder, immunosuppressive agents conceivably do not have any direct action upon the recovery of muscle function. It is plausible that the long period required for recovery of muscle strength may play a role in the weaning failure, emergency of infections, and the dismal prognosis of the disorder. Therefore, the use of a respiratory muscle training program could have a favourable impact on the outcome of the disease [9, 15, 16]. Indeed, the use of inspiratory muscle training has been found to be helpful in a variety of scenarios in which the mechanical ventilation weaning is troublesome [15, 17, 18].

We resorted to an isokinetic electronic load device using a protocol of endurance gain (higher number of repetitions using submaximal loads) given that the respiratory muscles display a large number of type I fibres, which are resistant to fatigue [9, 15, 16]. During the IMT program the patient showed no respiratory or hemodynamic changes that would prevent its continuation. The combination of the two strategies, one addressing the treatment of the acute immunological pathway and the other the recovery of muscle function, was associated with a successful weaning from mechanical ventilation, hospital discharge, and return to daily activities.

It is our view that the appropriate cleaning of the artificial airway, the correct positioning of the patient, and his collaboration were all essential to the success of the muscle training intervention.

In conclusion, the use of an inspiratory load device in association with the conventional therapy with immunosuppressive agents in the reported case was associated with an increase in the strength of the inspiratory muscles resulting in a favourable outcome. This is in line with previous reports above mentioned [15], but further studies are necessary to establish the exact role of IMT in the process of weaning from the ventilator these patients.

## Figures and Tables

**Table 1:** Compromise of muscular force in the lower limbs by Medical Research Council (MRC).

Muscles	Before treatment	After treatment
	Left/right	Left/right
Biceps brachii	3/3	5/5
Triceps brachii	1/1	4/4
Extensor carpi radialis	3/3	5/5
Iliopsoas	1/1	4/4
Quadriceps femoris	1/1	4/4
Tibialis anterior	2/2	4/4

Total	22	52

*MRC scoring system*. 0: no movement; 1: palpable contraction, no visible movement; 2: movement but only with gravity eliminated; 3: movement against gravity; 4: movement against resistance but weaker than normal; 5: normal power. [6].

**Table 2:** Evaluation of self-care, sphincters, and mobility by functional independence measure (FIM).

Parameters	Before treatment	After treatment
(1) Eating	1	7
(2) Grooming	1	7
(3) Bathing/showering	1	6
(4) Dressing upper body	1	6
(5) Dressing lower body	1	6
(6) Toileting	1	7
(7) Bladder management	2	7
(8) Bowel management mobility	2	7
(9) Transfers: bed/chair/wheelchair	1	7
(10) Transfers: toilet	1	7
(11) Locomotion: walking/wheelchair	1	7
(12) Locomotion: stairs	1	7

Total	14	81

*FIM scoring*. 7: complete independence, fully independent; 6: modified independence requiring the use of a device but no physical help; 5: supervision requiring only standby assistance or verbal prompting or help with setup; 4: minimal assistance requiring incidental hands-on help only (subject performs >75% of the task); 3: moderate assistance; subject still performs 50–75% of the task; 2: maximal assistance; subject provides less than half of the effort (25–49%); and 1: total assistance; subject contributes <25% of the effort or is unable to do the task [7, 8].
